# *Listeria monocytogenes* Behaviour in Presence of Non-UV-Irradiated Titanium Dioxide Nanoparticles

**DOI:** 10.1371/journal.pone.0084986

**Published:** 2014-01-09

**Authors:** Maria Grazia Ammendolia, Francesca Iosi, Barbara De Berardis, Giuliana Guccione, Fabiana Superti, Maria Pia Conte, Catia Longhi

**Affiliations:** 1 Department of Technology and Health, Istituto Superiore di Sanità, Rome, Italy; 2 Department of Environment and Primary Prevention, Istituto Superiore di Sanità, Rome, Italy; 3 Department of Public Health and Infectious Diseases, “Sapienza” University of Rome, Rome, Italy; Indian Institute of Science, India

## Abstract

*Listeria monocytogenes* is the agent of listeriosis, a food-borne disease. It represents a serious problem for the food industry because of its environmental persistence mainly due to its ability to form biofilm on a variety of surfaces. Microrganisms attached on the surfaces are a potential source of contamination for environment and animals and humans. Titanium dioxide nanoparticles (TiO_2_ NPs) are used in food industry in a variety of products and it was reported that daily exposure to these nanomaterials is very high. Anti-listerial activity of TiO_2_ NPs was investigated only with UV-irradiated nanomaterials, based on generation of reactive oxigen species (ROS) with antibacterial effect after UV exposure. Since both *Listeria monocytogenes* and TiO_2_ NPs are veicolated with foods, this study explores the interaction between *Listeria monocytogenes* and non UV-irradiated TiO_2_ NPs, with special focus on biofilm formation and intestinal cell interaction. Scanning electron microscopy and quantitative measurements of biofilm mass indicate that NPs influence both production and structural architecture of listerial biofilm. Moreover, TiO_2_ NPs show to interfere with bacterial interaction to intestinal cells. Increased biofilm production due to TiO_2_ NPs exposure may favour bacterial survival in environment and its transmission to animal and human hosts.

## Introduction

*Listeria monocytogenes* is an ubiquitous and opportunist pathogen that causes listeriosis, a deadly food-borne disease [1]. *L. monocytogenes* is mainly recognized as a problem for the food industry, due to its environmental persistence, attributed in part to its ability to form biofilms, that confer protection to bacterial cells and decrease the efficiency of cleaning and disinfection procedures [2] [3]. Microorganisms attached to a surface are an important potential source of contamination for any food material coming into contact with that surface [4] [5] [6].

Cases of listeriosis, that primarily affects pregnant women and immunologically compromised individuals, in recent years have increased in several European countries [7]. Listeriosis can manifest as gastroenteritis after ingestion of a high inoculum, as septicemia, meningitis and encephalitis primarily in immune-compromised individuals, and induce fetal-placental infection leading to in utero death, premature birth, abortion and neonatal infection. Moreover, *L. monocytogenes* can be also shed asymptomatically, persisting in human and animal faeces and be released in the environment [8] [9]. This bacterium also thrives in diverse external environments such as soil, water, decaying plants, and silage, exposing wild animals and cattle to multiple opportunities of ingestion and perpetuating *L. monocytogenes* transmission [10]. From a food-safety perspective and with the aim of limiting transmission to humans, a lot of emphasis has been focused on reducing bacterial aggregation, biofilm formation and persistence of bacteria on industrial surfaces and food [11].

In the last years, food industry has been made large use of particles as food additives. TiO_2_, also as nanoparticles (NPs), for example, is well appreciated for its inert capacities and as such widely used as a white food coloring compound, especially for confectionary, white sauces and dressings, and certain powdered foods [12] [13]. It is also used in the pharmaceutical industry as an opacity agent. Since TiO_2_ is inorganic, is highly stable and resistant to degradation. According to current European legislation, there is no maximum TiO_2_ level specified. It is recommended that TiO_2_ should be used in amounts according to good manufacturing practice [14]. The International Agency for Research on Cancer (IARC), after extensively studies on the hazardous potential, has rated TiO_2_ as possibly carcinogenic for humans (Group 2b) [15]. TiO_2_ are not declared in the contents of many products. Although daily intake is difficult to evaluate as dependent on the individual diet, a study by the European Food Safety Authorities (EFSA) estimated daily intake of TiO_2_ in 70–80 mg/day [16].

Titanium dioxide is typically found in gut tissue in the anatase polymorphic form. Nanoparticles may penetrate the intestinal barrier and accumulate in the mucosa. TiO_2_ particles have been shown to accumulate in M cells of Peyer's patches and are passed on to underlying macrophages [17]. *In vivo* studies on the capacity of TiO_2_ NPs to penetrate the gastrointestinal tract revealed that TiO_2_ could be found in systemic organs after an oral exposure of 10 days [18] [19]. TiO_2_ NPs have also been shown to be absorbed from the gastrointestinal tract (25, 80, and 155 nm; 5 g/kg BW; single oral dose; mice) [20]. In this study, TiO_2_ NPs were found cross the gastrointestinal tract through the lymphoid tissues surrounding it. However, since the dose used in this study was high, the extent of absorption under relevant human exposures is in question. More recently, Tassinari et al. (2013) [21] demontrated reproductive and endocrine effects of short term oral exposure to low doses (0, 1, 2 mg/kg BW per day) of TiO_2_ NPs.

Since this undegradability, TiO_2_ NPs are able to interact also with intestinal microrganisms or their products and induce inflammatory processes. As example, bacterial lipopolysaccharide (LPS), that is abundant in the gut, avidly binds TiO_2_ particle surfaces facilitated by calcium-bridging cations and mucosal secretions. It was observed that the complex induces release of proinflammatory citokines in primary human mononuclear phagocytes [22] or in intestinal explants [23].

During infection of the gastrointestinal tract, *L. monocytogenes* is in a particular environment with suboptimal conditions, including exposure to nanoparticles ingested with food. So far, the only studies about TiO_2_ NPs and *L. monocytogenes* concerned the photocatalytic activity of TiO_2_ NPs against bacteria, as alternative means of disinfecting surfaces or food contaminated by planktonic or biofilm bacteria [24] [25] [26] [27]. Since no data yet are available on the effects of non UV-irradiated TiO_2_ nanoparticles on listeria cells, the aim of present study was to explore interaction between bacteria and nano-sized TiO_2_, with special focus on biofilm formation and CaCo-2 cell colonization. Scanning electron microscopy was performed together with quantitative measurements of bacterial biofilm in presence of TiO_2_ NPs, to assess potential activity on bacterial cells. In addition, *in vitro* listerial cell interaction was evaluated, in order to explore nanoparticle influence on bacterial adhesion, invasion and intracellular replication.

## Methods

### Particle characterization

#### Sample preparation

Titanium (IV) oxide nanopowers (anatase <25 nm, 99.7%) were purchased from Sigma-Aldrich Company Ltd. (Gillingham, Dorset, UK)

Two milligrams of particles were weighted with a Mettler H54 AR electric balance (precision 0.1 mg) and suspended in culture medium in order to evaluate their behaviour in biological system.

Stock suspensions of nanoparticles were sonicated with a probe sonicator (Vibracell, 750 W, 20 KHz, amplitude 20%) in order to reduce agglomeration. Immediately after sonication, two millilitres of suspension were filtered through 0.05 µm pore polycarbonate membranes in order to reduce possible artifacts and aggregates of particles on TEM grid or SEM stubs that can be formed by transferring an aliquot of NP suspension.

#### Single particle characterization

The single nanoparticles were characterized by electron microscopy (scanning and transmission electron microscopy). For Scanning Electron Microscopy (SEM) analysis, portions of the polycarbonate filter were mounted on stubs and coated with a thin gold film deposited by sputtering. For Transmission Electron Microscopy (TEM) a thin carbon film was evaporated on the filter, then the polycarbonate was dissolved by chloroform.

Morphological analysis and primary size of NPs was performed by TEM (FEI EM208, FEI Company, The Netherlands) at an acceleration voltage of 80 KV.

A SEM (SEM FEI XL30, FEI Company, The Netherlands), equipped with Soft Imaging System, performed NP size distribution. The particles were automatically detected by an increase of the secondary electron video signal above a preset video threshold. More than 2000 particles were analysed and for each of them average diameter, aspect ratio and shape factor were determined. The main advantage of this system is to carry out statistical analysis on a large number of particles from data on the physical parameters selected.

From morphological analysis and size distribution we determined the agglomeration status of NPs in the bacterial culture medium.

### Bacterial strains and CaCo-2 cells

Two *L. monocytogenes* strains (LM2 and LM9) with different ability to produce biofilm were selected. Both strains were obtained from a collection of strains of Microbiology Institute, Department of Public Health and Infectious Diseases, “Sapienza” University of Rome.

Strains were biochemically controlled by the API *Listeria* kit (Bio Mérieux, France), according to the manufacturer's instructions. Haemolysis on Muller Hinton agar (Oxoid) supplemented with 5% sheep blood was used as additional test. Bacteria were maintained as stock cultures in 15% glycerol-brain heart infusion broth (BHI) (Oxoid) at −80°C.

LM2 and LM9 strains were previously characterised for biofilm formation and classified as moderate and strong biofilm producers, respectively [28].

Cells derived from a human colon carcinoma (CaCo-2) (ATCC® HTB-37) were purchased from American Type Culture Collection (ATCC; Manassas, VA, USA) and cultured in Dulbecco-modified minimum essential medium with Earle's salts (D-MEM, EuroClone), supplemented with 10% (v/v) heat-inactivated foetal calf serum (FCS, JRH Biosciences), and 2 mM glutamine. All incubations were carried out in a 5% CO_2_ atmosphere at 37°C. Cells were used 48 hrs after seeding.

### TiO_2_ NPs effect on bacteria

#### Bacterial growth assay

For preparation of inocula have to be tested with TiO_2_ NPs, *L. monocytogenes* strains were grown for 15 hrs in Tryptic Soy Broth (TSB) supplemented with 0.6% Yeast Extract at 37°C. Aliquots (1 ml) of diluted overnight cultures (OD 0.2) were mixed in a sterile test tube with TiO_2_ NPs at different concentrations (0, 0.08, 0.8, 8, 80 µg/ml) and incubated at 37°C with shacking (120 rpm). After 24 hrs incubation, bacterial cells were enumerated by colony forming units counts (CFU/ml) on Tryptic Soy Agar (TSA). Before inoculum, TiO_2_ NPs were suspended in bacterial medium and sonicated (mean potency/peak 90/180 W, +4°C) for 45 min to ensure dispersion of the particles.

#### Microtiter plate biofilm production assay

Overnight cultures of tested bacteria were diluted 1∶100 in fresh media. One hundred microliters of bacterial suspensions were added to each well in a 96-well plate containing 100 µl TiO_2_ NPs (sonicated as above) at different concentrations and incubated for 24 and 48 hours at 37°C in static conditions. The controls included a column of wells with each concentration of NPs in culture medium and TSB-YE without inoculum. Following incubation, the wells were washed five times with distilled water to remove non-attached cells and allowed to dry at 37°C for 1 hr. Then, were stained with 1% crystal violet (Sigma–Aldrich, St Louis, MO) for 30 minutes at room temperature. Stained wells were then washed five times with sterile distilled water, and the remaining crystal violet was eluted by the addition of 95% ethanol solution for 15 minutes. The biofilm biomass was then determined by measuring the absorbance at 590 nm. The microtiter plate biofilm assay was performed three times for both *L. monocytogenes* strains, and the averages and standard deviations were calculated for all repetitions of the experiment. All values were recorded after subctration of background absorbance of wells containing nanoparticles.

### Biofilm formation assay for scanning electron microscopy

Samples for scanning electron microscopy were prepared as follows: 24-well plates containing glass slides were inoculated with both *Listeria* strains in TSB-YE medium containing or not containing TiO_2_ nanoparticles at different concentrations. After 48 hrs incubation, samples were washed three times with phosphate buffered saline (PBS), fixed with glutaraldehyde 2.5% in 0.1 M cacodilate buffer (pH 7.4), and post-fixed in 1% OsO_4_ solution. After dehydratation in ethanol–water misture with increasing ethanol concentrations (65%, 75%, 85%, 95%, and 100%), biofilms were treated with hexamethyldisilazane (Sigma–Aldrich, St Louis, MO), and overnight air-dried. Dehydrated specimens were coated with a thin film of Au in a sputter coater. Morphological analysis was performed in an Ultra-high resolution Field Emission Gun Scanning Electron Microscopy (FEG-SEM, FEI Company). Secondary electron images were performed with an acceleration voltage of 10 KV. The images were processed for display using photoshop (Adobe Systems Inc., San Jose, CA, USA) software.

### Detection of cellular interaction of TiO_2_

The control and treated bacterial cells were prepared for SEM analysis as above described. In order to detect the TiO_2_ NPs on bacteria we used scanning electron microscopy equipped with a thin-window EDAX system for X-ray microanalysis by energy dispersion spectrometry. The electron beam energy was fixed at 25 KeV and EDX spectra were acquired in the range between 0 and 15 KeV.

### Adhesion, invasion and intracellular growth

The adhesion, invasion, and intracellular growth assays were carried out by infecting semiconfluent CaCo-2 cell monolayers grown in 24-well plates (Nalge Nunc International). Bacterial cultures containing logarithmically grown bacteria (MOI: 100 bacteria/cell) and TiO_2_ NPs at different concentrations were incubated with cells for 1 hr at 37°C. After this incubation period, cells were extensively washed, lysed with ice-cold 0.1% Triton X-100 and plated on TSA to determine bacterial adherence. Adhesion afficiency was expressed as the percent of the inoculated CFU that were recovered. The percentage of bacterial adhesion to CaCo-2 cells in presence of NPs was evaluated respect to the percentage of adhesion of *L. monocytogenes* in absence of NPs, considered as 100%.

For invasion assays, after washing, 1 ml of fresh medium containing 50 µg/ml of gentamicin was added to each well and maintained for 1 hr at 37°C. Cells were then lysed as for adhesion assay. Invasion efficiency was expressed as the percent of inoculated bacteria that were recovered. The percentage of invasion of bacteria to CaCo-2 cells in presence of NPs was evaluated respect to the percentage of invasion of listeriae in absence of NPs, considered as 100%.

For intracellular growth assays, incubation of cells in gentamicin-containing medium was prolonged for an additional period of 3 hrs at 37°C, followed by lysis and CFU counts. Intracellular growth was expressed as replication index (RI), corresponding to the number of CFU at 3 hrs post-infection divided by the number of CFU at time 0 (1 hr post infection).

### Statistical analysis

All results were calculated from data at least of three independent experiments and expressed as means ± Standard Deviation (SD). In all experiments, NP treated samples were compared with respective controls. Statistical analyses were performed by ONE WAY ANOVA (Graph Pad Prism, Version 5.0). A P value of less than 0.05 (P<0.05) was considered significant.

## Results

### Particle characterization and distribution

**Figure 1:**
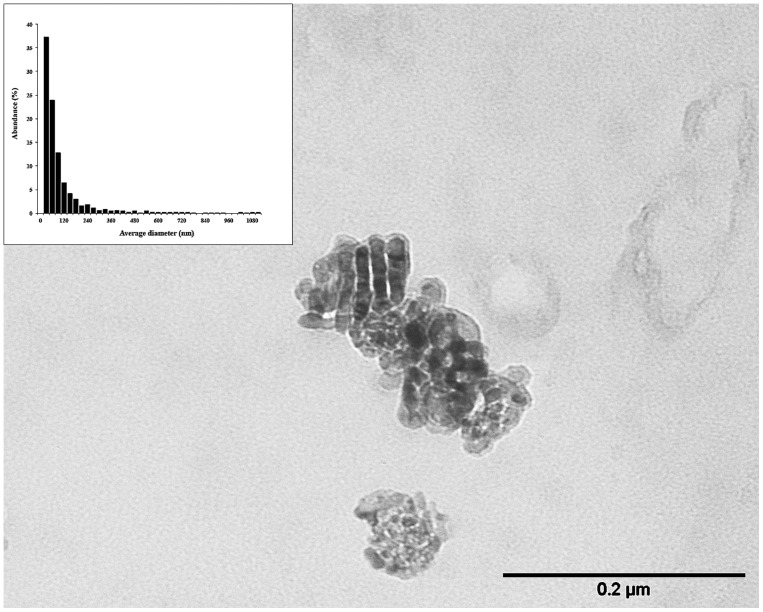
Transmission electron microscopy of TiO_2_ nanoparticles and size distribution performed by scanning electron microscopy. Single particle characterization was performed by filtering stock TiO_2_ NPs suspension on polycarbonate membranes. For TEM, samples were coated with a carbon film and then the polycarbonate was dissolved by chloroform. For SEM, filters, mounted on stubs, were coated with a thin gold film.

TEM analysis (Figure 1) showed that TiO_2_ NPs were spherules with primary size ranging from 20 nm to 60 nm. Occasionally, particles of irregular shape, mean length equal to 60 nm and mean width of 40 nm were observed. Size distribution, performed by SEM analysis (Figure 1, inset), showed that 98% of TiO_2_ NPs have a size ranging from 30 nm to 1.1 µm with a mean value equal to 139 nm; 61% of particles possessed dimensions less than 100 nm and 25% of them were aggregates between 100 nm and 200 nm. It is likely that, after the filtration, a percentage of particles less than 50 nm pore filters was not retained. In order to evaluate the percentage of particles not calculated in size distribution, we recovered the suspension after filtration and transferred it on silicon wafer. We estimated that the loss of particles less than 50 nm was 3%. This loss doesn't have a significant influence on the NPs size distribution because of the large number of particles analysed.

### TiO_2_ NPs effect on bacterial growth

**Figure 2:**
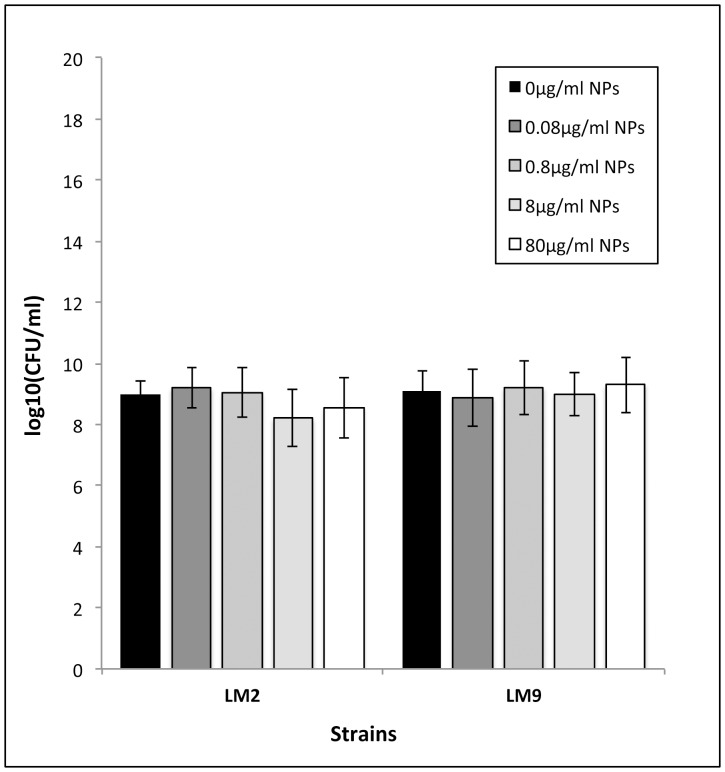
Effect of TiO_2_ NPs on growth of LM2 and LM9 strains. Bacterial cells were inoculated in TSB+YE supplemented with TiO_2_ NPs at concentrations of 0, 0.08, 0.8, 8, 80 µg/ml. Values are expressed as mean ± SD. All experiments were carried out in triplicate and repeated in two independent sets of experiments.

To evaluate nanoparticle toxicity, cell survival upon nanoparticle exposure was measured using count forming units assay. Data on the viability of both *L. monocytogenes* strains after exposure to TiO_2_ nanoparticles for 24 hours incubation period revealed no significant change in cell survival with increasing concentrations of TiO_2_ NPs (Figure 2).

### TiO_2_ NPs effect on biofilm formation

**Figure 3:**
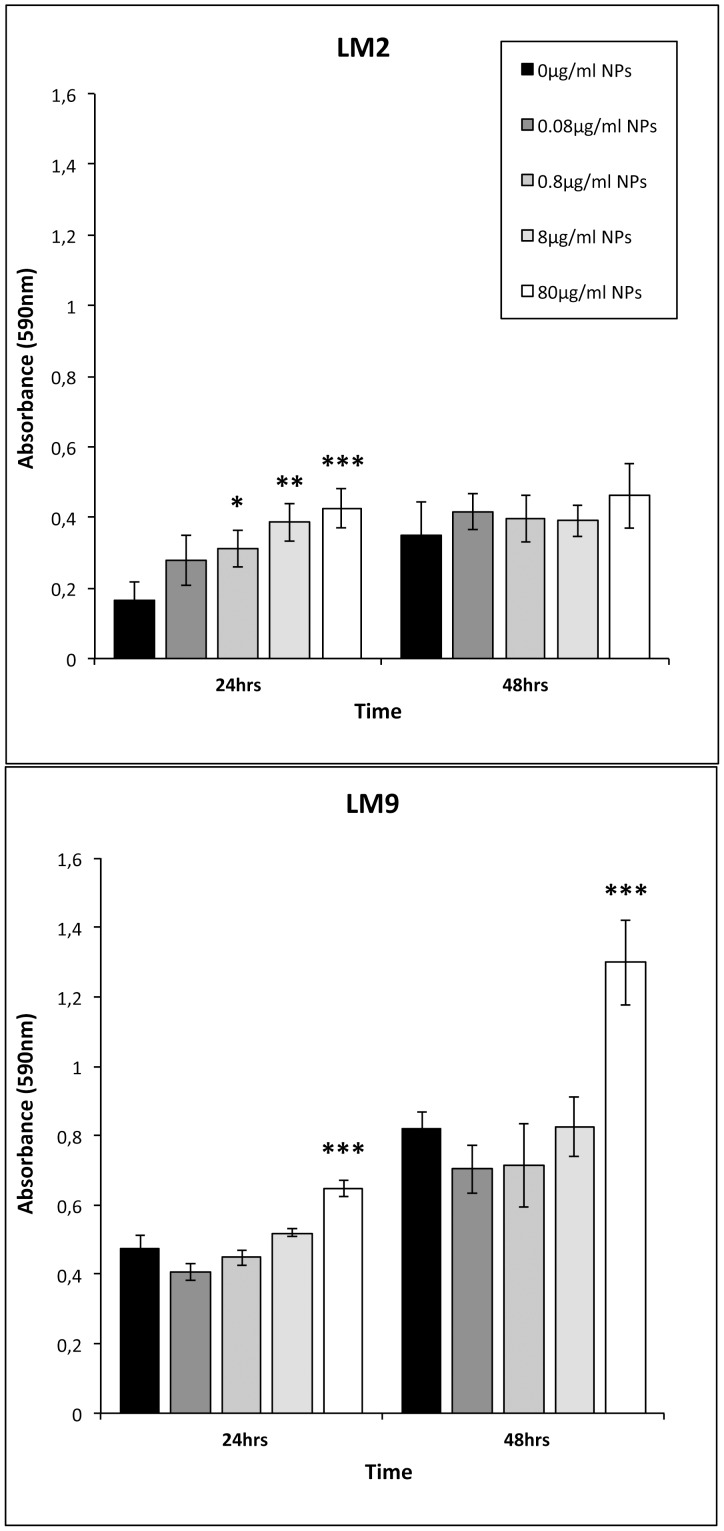
Effect of TiO_2_ NPs on biofilm formation by *Listeria monocytogenes* strains. Wells of polystyrene microtiter plates were conditioned with TSB-YE medium or with TiO_2_ NPs at 0.08, 0.8, 8, and 80 µg/ml. Biofilm cells were indirectly quantified by crystal violet staining and absorbance measurements at 590 nm. Values are expressed as mean ± SD. All experiments were carried out in sextuple and repeated in two independent set of experiments. Asterisks denote statistically significant increase of the *Listeria* population compared to control bacterial cells (*p<0.05; **p<0.01; ***p<0.001).

The influence of TiO_2_ NPs on capacities of *L. monocytogenes* strains to form biofilm was assayed under static conditions by crystal violet staining at 24 and 48 incubation hours. As shown in Figure 3, bacterial cultures without NPs are able to form a quantity of biofilm based on their biofilm production classification. The amount of biofilm generated by LM2, that is a moderate biofilm producer, resulted lower than those of LM9, a strong biofilm producer, both at 24 (OD590 nm = 0.165 for LM2 versus 0.473 for LM9) and 48 (OD590 nm = 0.24 for LM2 versus 0.819 for LM9) incubation hours. Biofilm mass formed in presence of TiO_2_ NPs appeared firstly increased in LM2 cultures, at all NPs doses utilised in a dose-dependent manner, during 24 incubations hours with significant values for 80, 8, and 0.8 µg/ml and then decreased to 48 hrs of incubation, with absorbance values comparable to control bacterial samples. Regarding LM9, only highest dose of TiO_2_ NPs induced increased biofilm mass at both 24 or 48 hrs with very significant values at later interval time.

### Scanning electron microscopy

**Figure 4:**
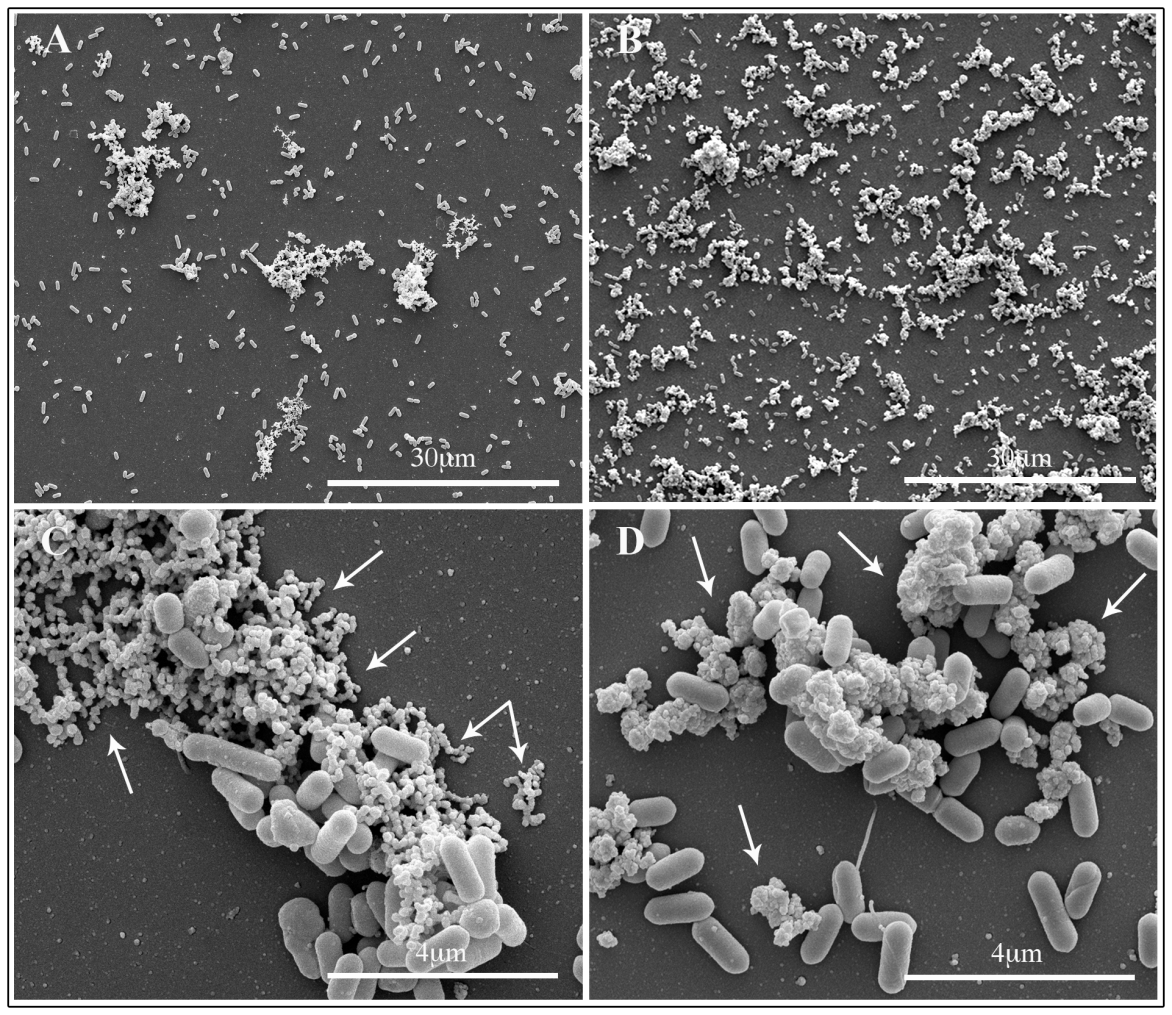
Scanning electron microscopy observations of LM2 strain biofilm formation. Bacteria were allowed develop biofilm on glass slide for 48_2_ NPs at different concentrations. Micrograph shows representative images of untreated (A–C) or 80 µg/ml TiO_2_ NPs (B–D) treated bacterial cells.

**Figure 5:**
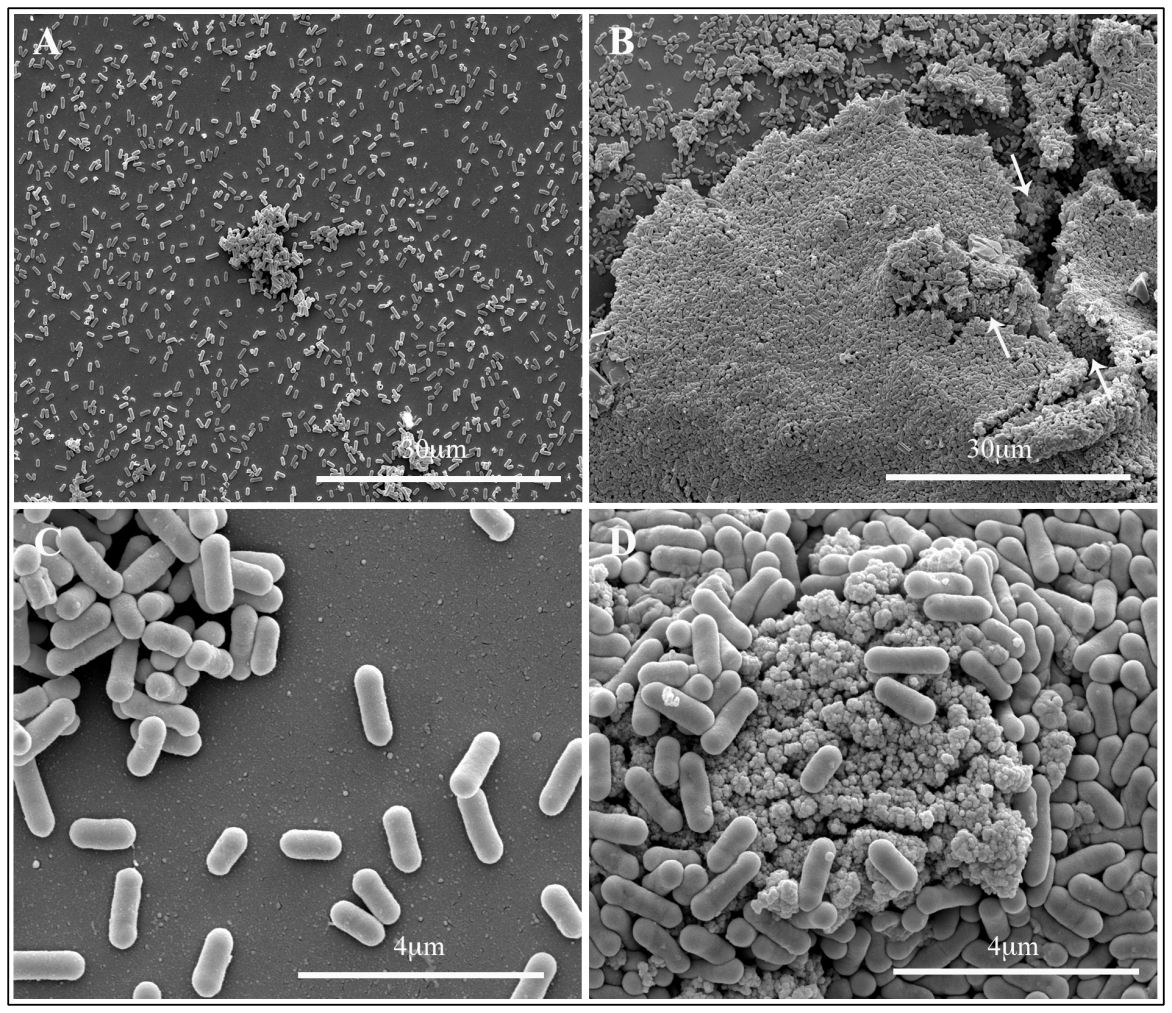
Scanning electron microscopy observations of LM9 strain biofilm formation. Bacteria were allowed develop biofilm on glass slide for 48_2_ NPs at different concentrations. Micrograph show representative images of untreated (A–C) or 80 µg/ml TiO_2_ NPs (B–D) treated bacterial cells.

Biofilm architecture of both *Listeria* strains with or without TiO_2_ NPs was examined after a 48 hrs incubation period. Representative micrographs by SEM of biofilm produced by LM2 and LM9 strains growth in presence of TiO_2_ NPs at 80 µg/ml dose are showed in Figure 4 and 5. Untreated LM2 biofilm appeared as isolated bacterial cells together with cell aggregates of small and medium size (Figure 4, panel A and C); an extracellular matrix, mainly on the aggregate cells, was also observed (Figure 4 C, arrows). Cells growth in presence of TIO_2_ NPs showed a carpet of bacteria, mainly aggregates, with threedimensional structure (Figure 4, panel B and D); nanoparticles appeared adherent to bacterial surface and seemed to hold bacteria together (Figure 4, panel D, arrows). LM9 biofilm mass without NPs showed most bacteria isolated or in medium size aggregates; the amount of extracellular matrix was much less or absent, compared to LM2 untreated biofilm (Figure 5, panel A and C). LM9 bacteria grown with NPs appeared with a architecture different from LM2. Biofilm mass was as large threedimensional structures composed by a lot of bacterial cells adherent to one another and on the nanoparticle surfaces. Also NPs formed large aggregates covered by bacteria with the aspect of mature biofilm structure (Figure 5, panel B, arrows); at higher magnification bacteria seemed either circumscribed with small nanoparticle clusters or are sorbed onto large nanoparticle agglomerates (Figure 5, panel D). Scanning observations of both *Listeria* strains grown with NPs confirmed data obtained with growth curves of NPs treated bacteria. Nanoparticle binding seemed not interfere with survival of adherent bacterial cells; most part of bacteria appeared structurally undamaged.

**Figure 6:**
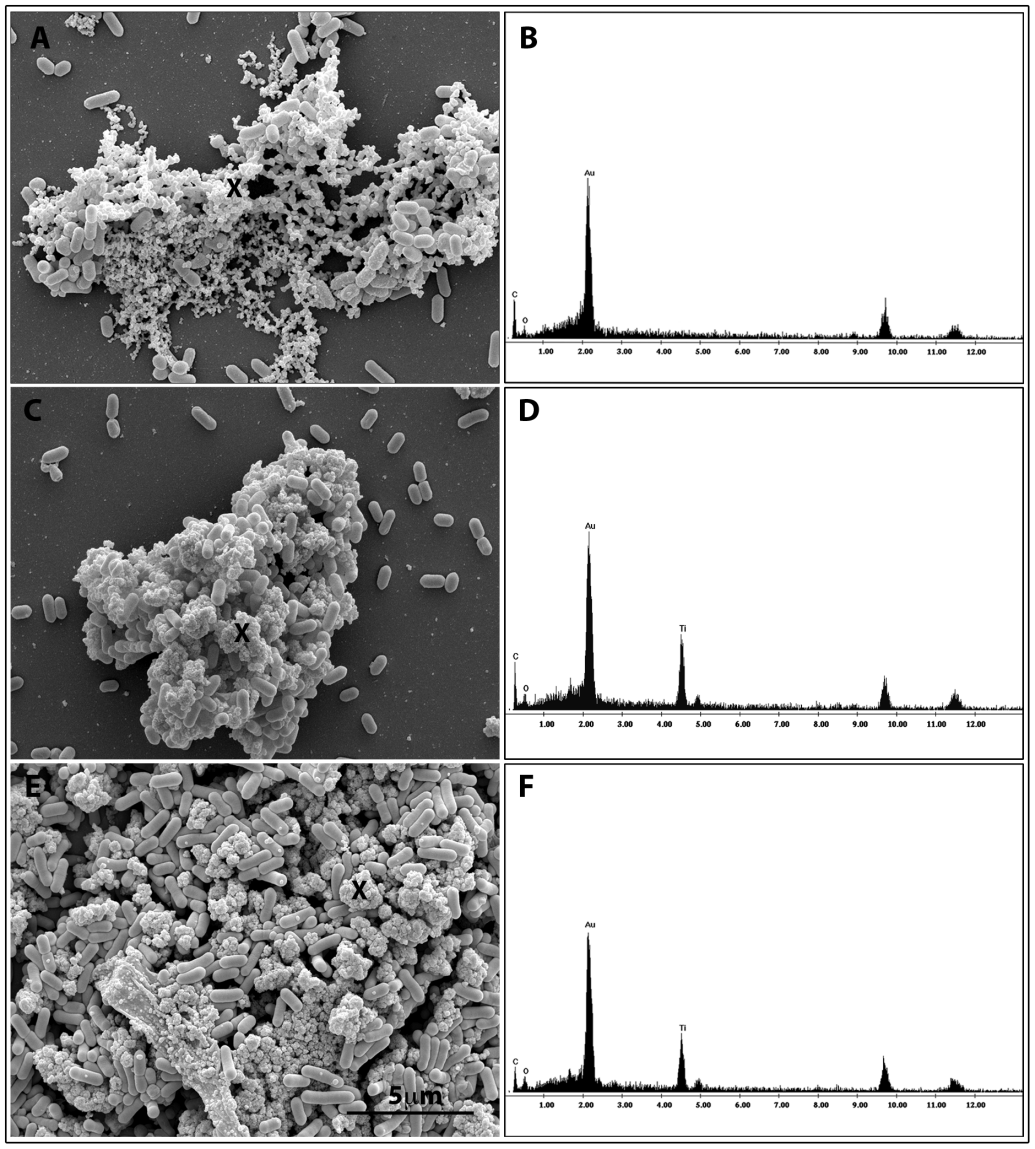
Scanning electron microscopy and energy-dispersive X-ray spectroscopic (EDX) analysis of LM2 and LM9 strains. LM2 untreated bacteria (A–B); LM2 strain exposed to 80 µg/ml TiO_2_ NPs (C–D); LM9 strain exposed to 80 µg/ml TiO_2_ NP (E–F). EDX spectra represent the qualitative elemental analysis of the extracellular matrix surrounding bacterial cells.

Surrounding matrix adherent to bacterial cells was investigated by X-ray microanalysis on clusters of bacterial biofilm grown in presence of nanoparticles. As showed in Figure 6, that was composed by representative micrographs of LM2 and LM9 exposed to NPs (panel C and E), Ti Kα and Ti Kβ peaks, O and C (panel D and F) were revealed differently to EDX spectra acquired on representative LM2 untreated bacteria (panel A) showing only C and O peaks (panel B).

### TiO_2_ NPs effect on bacteria-cell monolayers interaction

**Figure 7:**
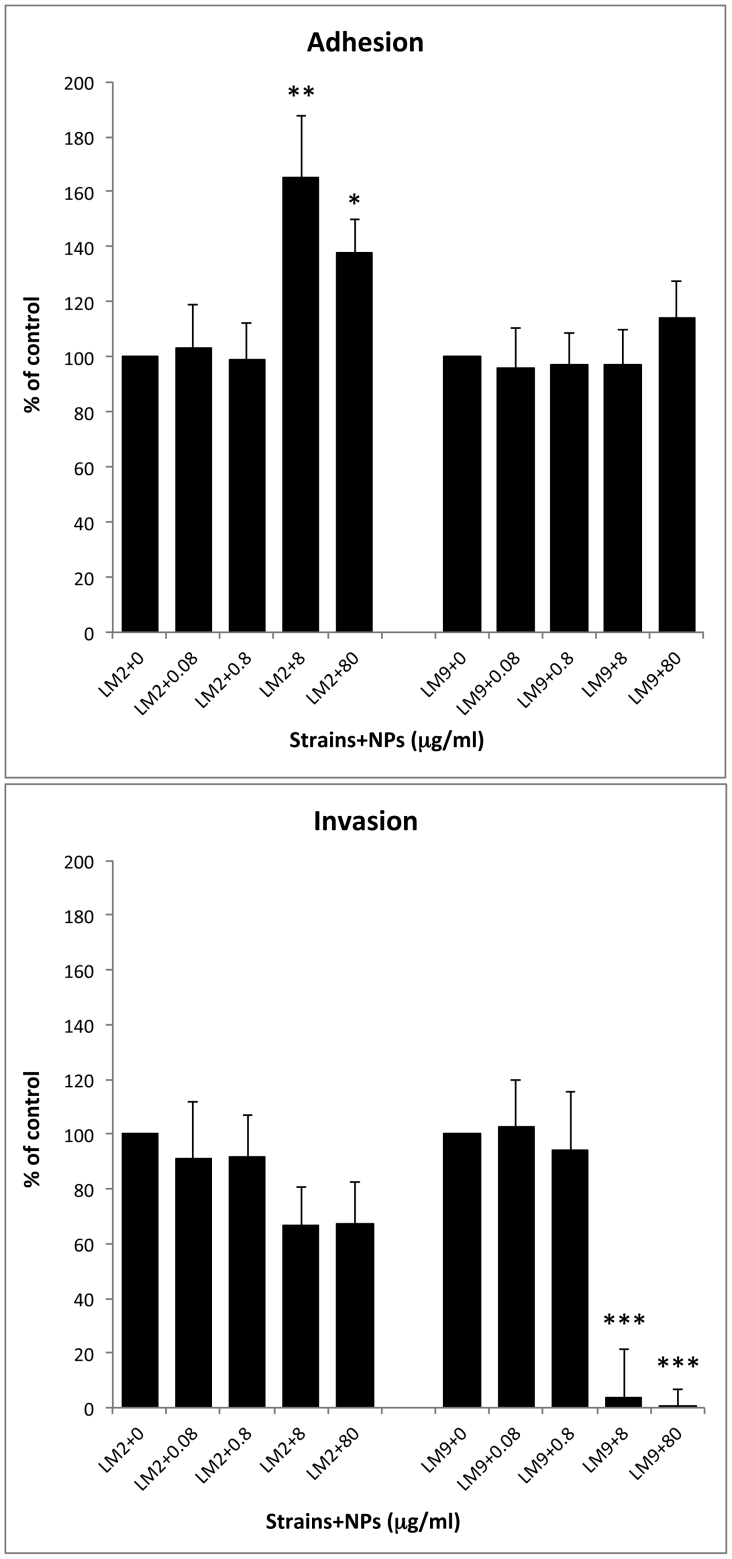
CaCo-2 adhesion and invasion efficiency of LM2 and LM9 strains in absence or in presence of TiO_2_ NPs at different concentrations. Values were expressed as percentage of control untreated bacteria. Both adhesion and invasion were performed in triplicate and three independent experiments were carried out for each strain. Asterisks denote statistically significant values compared to control bacterial cells (*p<0.05; **p<0.01; ***p<0.001).

The capability of LM2 and LM9 bacterial cells, grown in TiO_2_ NPs supplemented media, to adhere, enter and multiply in CaCo-2 cells was assayed (Figure 7). When LM2 was grown with higher NPs doses, the percentage of adherent bacteria in CaCo-2 cells was significantly enhanced respect to control bacterial cells (165.1%±22.7 at 8 µg/ml NPs and 137.93%±12 at 80 µg/ml) whereas low NPs doses were ineffective. Differently, LM9 adhesion appeared unaffected by bacterial growth at all NPs doses utilised. Regarding bacterial invasion, notwithstanding better adhesion, LM2 grown with 8 and 80 µg/ml of TiO_2_ NPs showed a reduced internalization by CaCo-2 cells (66.6±14.15 and 67.5±14.8). Decrease of internalization was more pronounced for LM9 strain; at higher doses of TiO_2_ NPs bacterial invasion was dramatically reduced (3.54%±17.96 for 8 µg/ml and 0.44%±6.17 for 80 µg/ml) (p<0.001). As for LM2, lower doses were not able to influence also LM9 entry to CaCo-2 cells.

The capability of *L. monocytogenes* strains to multiply in human intestinal-like CaCo-2 cells is reported in Table 1. The intracellular multiplication values, express as replication index at 3 hours post-infection, showed that, as already demonstrated (Longhi et al. 2003), LM9 strain was able to multiply in cell monolayers with higher efficiency respect to LM2 isolate (11.12±0.2 and 1.55±0.1, respectively). Interestingly, 8 µg/ml of TiO_2_ NPs was already sufficient to half reduce intracellular multiplication of LM2 strain; to obtain the same half inhibition with LM9 strain, a ten higher concentration was required.

**Table 1:** Replication index (RI) of listeria strains in CaCo-2 cells in presence of TiO_2_ NPs.

Strains + Ti0_2_ NPs (µg/ml)	Replication Index
LM2+0	1.55±0.1
LM2+0.08	1.45±0.3
LM2+0.8	1.22±0.4
LM2+8	0.8±0.3
LM2+80	0.75±0.4*
	
LM9+0	11.12±0.2
LM9+0.08	11.4±0.3
LM9+0.8	11.2±0.5
LM9+8	10.57±0.3
LM9+80	5.41±0.1***

Replication index: Intracellular growth was expressed as replication index (RI) which corresponds to the number of CFU 3 hrs post-infection divided by the number of CFU 1 hr post-infection. Data are presented as means (± standard deviation) of at least three experiments. Asterisks indicate statistically significant values compared to control bacterial cells (*p<0.05; ***p<0.001).

## Discussion

Studies about interaction between *Listeria monocytogenes* and TiO_2_ nanoparticles concerned antibacterial activity due to photocatalytic effect of UV-irradiated nanoparticles. Antimicrobial mechanism seems to be due to generation of free electrons and electron vacancies, or holes, with consequent production of reactive oxygen species (ROS), following excitation of nanoparticles by UV or near-UV irradiation [29].

To our knowledge, the influence of TiO_2_ NPs on *Listeria* bacterial cells in absence of UV irradiation was not reported so far. We explored this condition since large use of non UV-irradiated TiO_2_ NPs in a variety of commercial products included foods and their possible concomitant presence with *L. monocytogenes* in gut.

Differently to that observed for TiO_2_ NPs antibacterial effect on some bacterial species in solar light [30], interaction between TiO_2_ NPs and *Listeria monocytogenes* doesn't appeared decrease listerial vitality. Antibacterial activity has been attributed to disturbance of the cell membrane and osmolarity [31] [32] and the efficiency of TiO_2_ NPs action, observed also in the dark, seemed due to close contact of NPs with the bacterial cell surface [33] [34]. Regarding *Listeria monocytogenes* LM2 and LM9 strains, close contact with NPs was extensively observed by scanning electron microscopy but these interactions were not able to influence bacterial growth. Biofilm formation, on the contrary, resulted increased at higher NPs concentrations with difference related to intrinsic capability of each strain to produce biofilm. Most effect was revealed on the strong biofilm producer LM9 that showed a significant value of absorbance with the highest NPs concentration. Scanning images provided a picture of biofilm mass with large threedimensional structure in which bacterial cells appeared adherent to one another and on the nanoparticle surface. It is known that the hydrophobicity of both bacteria and surfaces could play a role in initial interaction each other in dependence of several factors such as temperature, nutritional contents or bacterial features [35]. Some Authors reported that *Listeria monocytogenes* cell surfaces were found to be hydrophilic when bacteria were grown at 37°C in TSB supplemented with yeast extract, the our experimental culture conditions, and that the bacterial hydrophobicity increased as pH drops [36] [37]. In our biofilm assays, after 2 incubation hours, the pH of culture dropped to 5.5 remaining unchanged during the time of assay (data not shown). Then, the successively increased hydrophobicity and the high negative charge that characterize, as reported by Briandet et al. (1999) [37], *L. monocytogenes* under definite growth conditions, could be responsible of close adhesion of bacterial surface to NPs. In fact, the TiO_2_ NPs of many commercial preparations have an isoelectric point at pH 6.0–7.0, and their surface is negatively charged at pH>7 and positively charged at pH<6 [38] [39] [40].

NPs binding could involve several bacterial surface proteins, such as internalins (A and B) or ActA. Regarding ActA, that it is known to mediate actin-based intra- and intercellular spread, some authors [41] demonstrated that it is also critical for bacterial aggregation and biofilm formation. ActA is attached to the bacterial surface via a highly hydrophobic C-terminal membrane anchor and promotes *Listeria monocytogenes* aggregation via direct ActA-ActA interaction. Since ActA is present on the entire bacterial surface and promote biofilm formation, it could contribute also to increased biofilm formation by NPs. It has been demonstrated that ActA-mediated aggregation is maximal between pH 6.5 to pH 9.0 [41]. At pH 5.5, our experimental biofilm conditions, ActA-ActA interactions were no maximal, consequently TiO_2_ NPs could bind ActA free proteins and contribute to aggregation, providing an additive mechanism of increasing bacterial biofilm.

Results obtained by gentamicin protection assay indicated that the higher concentrations of nanoparticles produced a reduction of invasion capability for both *L. monocytogenes* LM2 and LM9 strains. *In vitro* studies have demonstrated that *Listeria* enters into nonphagocytic cells through a zippering process and invasion proteins Internalin A and B are involved [42] [43]. Upon internalization, the bacterium is entrapped in a membrane bound compartment, from which it is able to escape [44] [45] through the action of the listeriolysin O (LLO) protein [43] [46]. Upon vacuolar lysis, *L. monocytogenes* reaches the cytosol, in which it can polymerize host actin to form comet tails that propel bacteria intracellularly [47].

The decrease of bacterial internalization in cultured cells observed in the presence of TiO_2_ NPs suggests that their binding to several bacterial proteins, such as internalins or ActA, that it has been demonstrated also play a role during entry process [48], could be involved. LM2 and LM9 strains showed both the full-length form of ActA [49] and the different behaviour in invasion capability could be linked to a complementary possible role of internalins or a different expression level of *ActA* genes, as suggested by Travier et al. (2013) [41]. During bacterial cell infection the main ActA-mediated aggregation observable at pH 6.5 could explain the behavior of LM9 strain in which a strong invasion inhibition was revealed with higher NPs concentrations. At this pH bacteria mainly aggregate via ActA and became less able to invade epithelial cells.

Different behaviour between LM2 and LM9 strains was found also regarding intracellular multiplication in CaCo-2 cells. The higher NPs dose required to half reduce LM9 replication index could be explained through different expression levels of LLO. The more elevated haemolytic property showed by LM9 respect to LM2 strain [49] could influence the activity of TiO_2_ NPs regarding vacuolar escape.

Altogether our experiments demonstrate that exposure to non UV-irradiated TiO_2_ nanoparticles resulted in increased biofilm formation by *Listeria* and reduced invasion and intracellular multiplication. The influence of NPs appeared correlated with intrinsic features of listeria strains and environmental conditions. Permissive pH found in some gut districts may favour close contact between NPs and bacteria; as bacterial-NPs aggregation occurs at pH lightly acidic, *L. monocytogenes* could be subjected to a pH permissive to binding of nanoparticles in duodenum and cecum lumens. This biofilm favouring property by TiO_2_ NPs correlates both with more efficiently colonization of gut and fecal shedding. Biofilm growth may protect bacteria against host defenses and the action of antimicrobial agents but also planktonic cells may be continuously shed from the biofilm to re-infect the same host or be transmitted. Moreover, the release of listerial aggregates, as opposed to isolated bacteria, may favor bacterial survival in environment and its transmission to animal and human hosts [50] [51]. Nanoparticles also, entraped inside bacterial biofilm, may induce inflammatory responses towards bacterial antigens, acting as adjuvant as reported by Butler et al. (2007) [52], or increase secretion of pro-inflammatory cytokines by conjugating to lipopolysaccharide present in intestinal lumen [22] [23].

Reduced invasivity and cellular multiplication with higher doses of NPs could induce invoke a defensive activity provided by nanoparticles against *Listeria*. Although this hypothesis cannot be excluded, it is also likely that NPs at low doses can be transported more easily through the intestinal barrier by listeria cells; a rapid trancytosis of *L. monocytogenes* through M cells, a process that occurs independently from the action of classical virulence factors, it has been demonstrated [53].

## Conclusions

Overall, results of the present study report new insights onto interaction between non UV-irradiated TiO_2_ nanoparticles and bacteria ingested with food and possible risks for environmental listeria spreading and trasmission to animals and humans. Increased studies should be targeted to the possible role of TiO_2_ NPs on bacterial agents transmitted with foods taking into account non-catalytic activity and possible modulation of virulence factors. Since TiO_2_ NPs intake has been estimated on the order of 1 mg per kilogram body weight per day [54], it is reasonable to hypothesize that prolonged exposure to high doses of NPs could induce the effects we observed on *Listeria monocytogenes*. For this, possible enhanced bacterial virulence should be carefully considered in risk assessment of nanoparticles present in food. Further studies will be focused on bacterial surface proteins affected by TiO_2_ NPs binding and mechanisms involved in bacterial infection in presence of NPs.
